# The Neural Basis of Social Cognition in Typically Developing Children and Its Relationship to Social Functioning

**DOI:** 10.3389/fpsyg.2021.714176

**Published:** 2021-12-10

**Authors:** Sarah Hope Lincoln, Cora M. Mukerji, David Dodell-Feder, Arianna Riccio, Christine I. Hooker

**Affiliations:** ^1^Department of Psychological Sciences, Case Western Reserve University, Cleveland, OH, United States; ^2^Department of Psychology, Bryn Mawr College, Bryn Mawr, PA, United States; ^3^Department of Psychology, University of Rochester, Rochester, NY, United States; ^4^Department of Neuroscience, University of Rochester Medical Center, Rochester, NY, United States; ^5^Boston University School of Social Work, Boston, MA, United States; ^6^Department of Psychiatry and Behavioral Sciences, Rush University Medical Center, Chicago, IL, United States

**Keywords:** social cognition, social behavior, developmental psychology, developmental cognitive neuroscience, theory of mind

## Abstract

Theory of mind (ToM), the ability to think about the perspectives, beliefs, and feelings of another, develops throughout childhood and adolescence and is an important skill for social interactions. This study examines neural activity in typically developing children during a novel ToM task – the Movie Mentalizing Task– and tests its relations to ToM behavioral performance and social functioning. In this fMRI task, children ages 8–13years (*N*=25) watched a brief movie clip and were asked to predict a character’s mental state after a social interaction. Engaging in the Movie Mentalizing Task activated the ToM neural network. Moreover, greater neural activity in the ToM network, including the superior temporal gyrus and inferior frontal gyrus, was associated with better behavioral performance on independent ToM tasks and was related to better social functioning, though these results do not survive correction for multiple comparisons. Results offer a new affective theory of mind task for children in the scanner that robustly recruits activity in theory of mind regions.

## Introduction

Theory of mind – the ability to reason about others’ mental states – is critical for effective social interaction and communication. Social-cognitive abilities like theory of mind (ToM) in childhood are associated with typically developing children’s adaptive social behavior (Caputi et al., 2012; Fink et al., 2015b; Devine et al., 2016). Deficits in social-cognitive ability are associated with negative outcomes including peer rejection and bullying in typically developing children (Banerjee et al., 2011; Shakoor et al., 2012). Understanding the neurobiological mechanisms that support these processes may offer important insight into typical and atypical patterns of social functioning among children. Only a handful of studies (e.g., Richardson et al., 2018; Mukerji et al., 2019) have bridged the gap between the neurobiology supporting ToM processing and social functioning in children. The following study addresses this gap in the literature by investigating the neural basis of ToM in typically developing school-aged children and the relationship among ToM-related neural activity, ToM behavioral performance, and broader aspects of social functioning. We examined these brain-behavior relationships using a novel fMRI theory of mind task specifically designed for neuroimaging studies with children.

There is a developmental progression of ToM abilities throughout early childhood into adolescence. Early in development, research suggests that infants and toddlers can make connections between physical cues, such as gaze or facial expressions to behaviors related to perception or emotions (Phillips et al., 2002; Saxe et al., 2004). Typically developing children, by ages 4 and 5, are then able to use that mental representation to reason and make predictions about a person’s beliefs, goals, desires, emotions, and motivations (Saxe et al., 2004). Language plays a particularly important role in this shift at ages 4 and 5. Developmentally, there is a specific shift in more complex theory of mind tasks between the ages of 3–5 years; most 3-year-old children will fail a false belief task, that 5-year-old children can successfully complete (Wellman et al., 2001). However, there is some research to suggest that this developmental change may be linked to the development of general cognitive processing abilities (including language and executive functioning) that allow children to explicitly reason about false beliefs, rather than a developmental transition specific to social-cognitive abilities, as some literature suggests that infants and toddlers may be able to understand false belief (Baillargeon et al., 2010; Scott and Baillargeon, 2017). Additionally, though children demonstrate a mastery of many types of ToM skills [e.g., false belief reasoning (Miller, 2009)] by elementary school age, research suggests that ToM continues to improve in middle childhood (Devine and Hughes, 2013) and that use of theory of mind increases from early to late adolescence (Dumontheil et al., 2010), a finding congruent with neuroimaging studies showing continued maturation of brain regions involved in ToM processing (Blakemore, 2008; Vetter et al., 2014; Bowman et al., 2019).

Research with adults has identified a network of regions, including the medial prefrontal cortex (MPFC), anterior cingulate cortex, precuneus, right superior temporal sulcus, bilateral temporal poles, bilateral temporoparietal junction (TPJ), and inferior frontal gyrus (IFG), which support theory of mind processing (Schurz et al., 2014; Molenberghs et al., 2016). Though previous work has shown that during ToM tasks typically developing children recruit these same regions that have been identified in adults (Kobayashi et al., 2007; Saxe et al., 2009), there remain important developmental changes in these ToM regions. Specifically, studies suggest that there is an increased functional specialization of ToM regions between the ages of 3–12years in typically developing children (Gweon et al., 2012; Richardson et al., 2018).

Several research studies have shown that individual differences in children’s ToM ability, as measured on behavioral tasks, are related to differences in social functioning. Research shows that in typically developing children and deaf children ages 5–13, ToM ability was found to be a more reliable predictor of overall social skills than gender, age, or language proficiencies (Peterson et al., 2016). Caputi et al. (2012) found that ToM was positively related to teachers’ ratings of typically developing children’s prosocial behavior over multiple time points. Furthermore, ToM is especially salient within friendship formation and interpersonal relationships in typically developing children (Banerjee et al., 2011; Devine and Hughes, 2013; Fink et al., 2015a,b). A potential rationale for such outcomes lies within ToM development such that a more accurate understanding of another’s mental state could facilitate more harmonious relations *via* increased communication, cooperation, and general rapport (Peterson et al., 2015).

To date, research has identified a network of regions reliably active during ToM tasks and, presumably, responsible for thinking about the mental states of others (e.g., Gweon et al., 2012). Additionally, studies have found that behavioral measures of ToM are related to real-world social functioning (e.g., Peterson et al., 2016). What remains less well understood is the brain-behavior connection, linking ToM-related neural activity, ToM or social-cognitive ability, and social functioning, particularly in school-aged children. Understanding the relationship between the neurobiology of social-cognitive processes like ToM and social functioning could provide important insight into typical social development and atypical social development, such as the ToM processing deficits seen in individuals with autism spectrum disorders (Kennedy and Adolphs, 2012). The following study addresses this gap in the literature, investigating the relationship among neural activity in a novel ToM task, behavioral measures of ToM, and social functioning.

In this study, we use a novel ToM task: the Movie Mentalizing Task. In contrast to a false belief task, which is a cognitive theory of mind task, the Movie Mentalizing Task is an affective, or emotion inferencing, task. ToM tasks, such as the false belief paradigm, have come under scrutiny regarding the general/non-ToM cognitive demands (e.g., attentional, linguistic, executive control) that may better account for differences in performance on this task (Bloom and German, 2000; Perner and Leekam, 2008). One aim of this study was to pilot the use of a novel task which corrects for these problems. While watching the movie, an activity that children have likely done many times before, we ask them to follow the story and predict what happens next. Children do not need to reason about a belief that is false, an inherent difficulty in false belief tasks (Bloom and German, 2000). The differences in our task relative to previous ToM tasks allow us to gain a better understanding of how children think about more real-life social interactions. While children are not engaged in the interaction themselves, a movie may allow greater immersion into the social situation, giving us data that move us closer to understanding children’s knowledge of social interactions in the real world, while in the scanner. Additionally, our control condition specifically isolates theory of mind processing relative to general social information; in the ToM condition, children are asked to predict what a character may do next, following the video clip, whereas in the social information (control) condition, children still attend to social information by counting the number of people in the video clip. There have been several previous theory of mind tasks that rely on passive viewing or listening, of mental vs. physical states, in a movie (Richardson et al., 2018; Richardson and Saxe, 2019) or story (Saxe et al., 2009) paradigm. These tasks do not have the cognitive demands of a false belief task. However, these tasks are passive and do not prompt the child to engage in mentalizing. In our task, participants are given specific instructions prior to the video clip to pay attention to a specific person and then are asked in the response to determine what emotional behavior (e.g., cry) that character might do next. Saxe (2006) describes the importance of language or verbal communication in real-life social conditions, indicating that ToM tasks that are most appropriate or best target the processes individuals use in day-to-day life will involve reasoning about third-person situations in which verbal communication is the primary modality. By asking participants to reason about a movie character’s mental state based on both verbal and nonverbal communication between characters, the Movie Mentalizing Task more accurately approximates how children might use social cognition in day-to-day life. Furthermore, films are beneficial to use with child participants in neuroimaging studies because they keep participants focused, entertained, and even limit movement within the MRI scanner (Richardson et al., 2018). Thus, this task improves upon prior ToM tasks and offers the field a new ToM task appropriate for use in the scanner with children.

The current study has two primary objectives. First, we investigated the neural networks involved in theory of mind in children using a novel pediatric fMRI task. Second, we tested the relationship between neural activation and behavioral measures of social cognition and social functioning, to identify brain-behavior relationships. We hypothesized that this new task will tap into core regions in the affective ToM network. In addition, given that previous research has demonstrated a strong relationship between social cognition and social functioning, we hypothesized that a relationship between neural activity in ToM regions will relate to behavioral ToM performance and social functioning. We anticipated that the same regions that support theory of mind processing would support social functioning as well.

## Materials and Methods

### Participants

Forty-three children ages 8–12years old from the greater Boston community were recruited to participate in this study. Exclusion criteria included left-handedness, an IQ<70, and a past or present psychiatric disorder as assessed by the Kiddie Schedule for Affective Disorders and Schizophrenia (KSADS; Kaufman et al., 2000). Sixteen children were excluded from the study for excessive movement during the scan, defined as more than 20% of volumes being >2mm from the previous image or +/− 2.5 SD from the global mean intensity signal. Two children were removed from the sample due to technical issues (e.g., scanner malfunction). The final sample included 25 participants. We obtained consent from all guardians and assent from all participants. For taking part in the study, the child participants and parents were compensated with gift cards. The procedures for this study were approved by the Harvard University Institutional Review Board, study 21305.

### Behavioral Tasks

Participants completed measures for this study over the course of two study visits. During the first session, child participants completed a K-SADS and a Wechsler Intelligence Scale for Children (WISC)/Wechsler Abbreviated Scale of Intelligence (WASI) in addition to two ToM tasks: the Hinting Task (Schenkel et al., 2008) and the Reading the Mind in the Eyes task (RMET; Baron-Cohen et al., 2001b). Parents or guardians completed a parent report measure of their child’s social functioning using the Social Skills Improvement System Rating Scales (SSIS; Gresham and Elliott, 2008). During the second visit, child participants completed a mock MRI scan in addition to three practice trials of the Movie Mentalizing Task to ensure children’s understanding of the task. Upon successful completion of these trials, child participants were scanned while completing the Movie Mentalizing Task. Parents or guardians did not complete any additional tasks during the second session.

#### Hinting Task

The Hinting Task (Schenkel et al., 2008) consists of 10 short scenarios describing a social interaction in which a character uses subtle verbal and social cues to hint at something they need from another character. Children are asked to infer the meaning of the hint. If the child fails to accurately infer the hint, they are asked a follow-up question, which provides an additional clue. Children are given two points for a correct answer on the first try, one point for a correct answer when given the clue and no points if they fail to accurately infer the hint. There are 10 questions for a total of 20 possible points. The task was originally created for an older age group and was modified for children by Schenkel et al. (2008) to assess ToM in children and adolescents with bipolar disorder; we used the modified version in this study.

#### Reading the Mind in the Eyes Task

Children completed the child and adolescent version of the RMET (Baron-Cohen et al., 2001a). This task requires participants to look at pictures of people’s faces and infer their emotional states by the expression of their eyes. Children are presented with 28 pictures with four possible choices for each picture; the child receives one point for each correct response, for a possible 28 total points. The RMET has been used with individuals with autism spectrum disorder, schizophrenia, personality disorders, and traumatic brain injury (Baron-Cohen et al., 2001b; Bora et al., 2009; Fertuck et al., 2009).

#### Social Skills Improvement System Rating Scale

The SSIS-RS (Gresham and Elliott, 2008) can be used with multiple raters (child, parent, and teacher) to assess three major domains of children’s development: social skills, problem behaviors, and academic competence. In this study, we used parent rated measures to assess social skills. This assessment is a measure of social functioning. The social skills domain assesses the following aspects of children’s social development: communication, cooperation, assertion, responsibility, empathy, engagement, and self-control. The SSIS-RS has been developed for use with children ages 3–18, with specific tests for 5- to- 12-year-old children and 13- to 18-year-old children. Parents are asked to rate how true a statement is on a 4-point scale of *not true*, *a little true*, *a lot true*, and *very true*; higher scores reflect better social functioning.

#### Theory of Mind Task: Movie Mentalizing Task

**Figure 1:**
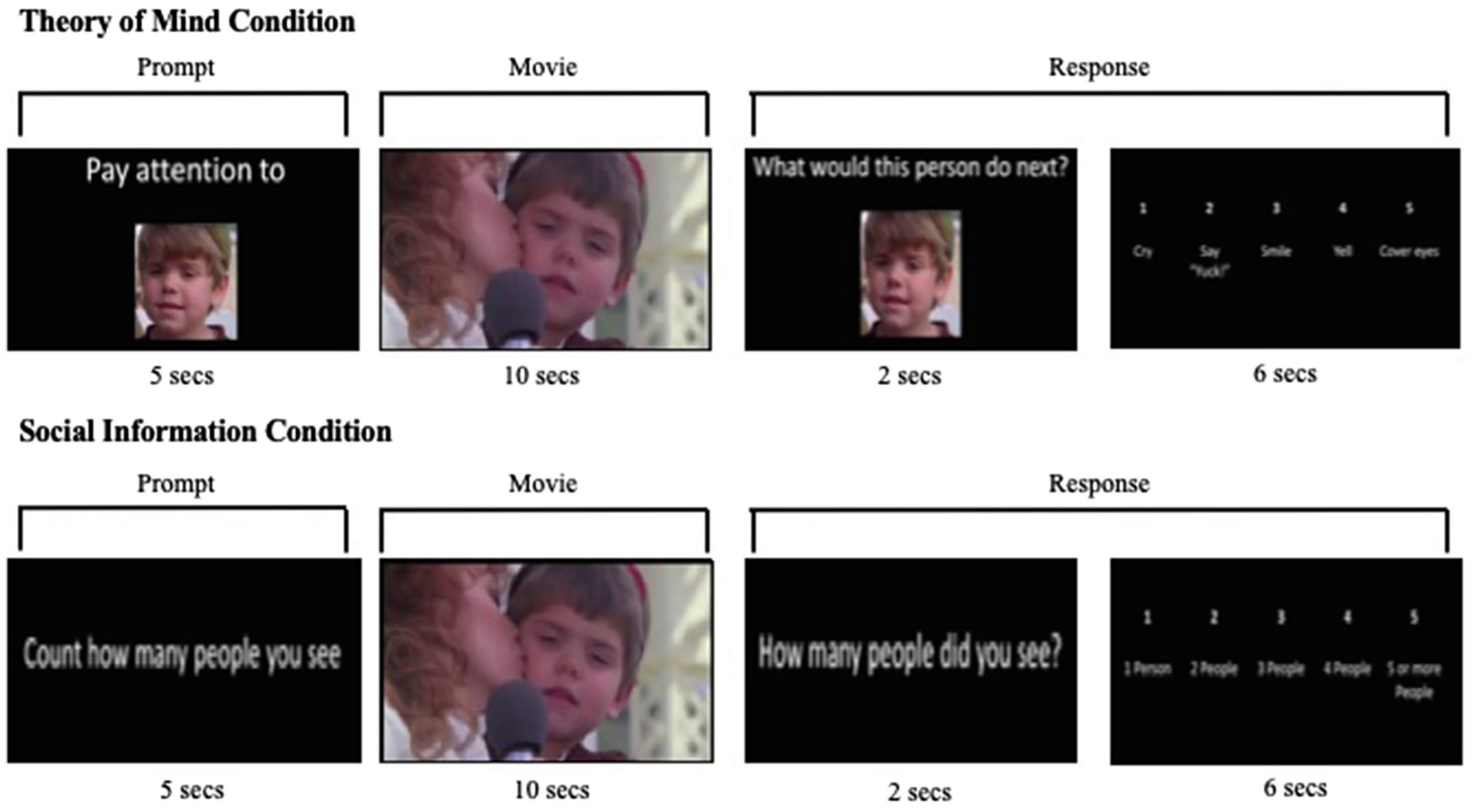
Movie Mentalizing Task.

The Movie Mentalizing Task (Figure 1) is a novel ToM task designed for use in the scanner. Specifically, this task asks participants to mentalize – that is, to infer and reason about mental states from human actions – and then use that inference to predict subsequent behavior. The Movie Mentalizing Task uses the 1994 film *The Little Rascals* and five of its main characters to assess children’s brain function, as they watch movie clips and make decisions about what a character would do next (theory of mind condition) or how many people were in a scene (social information condition).

For this task, each trial consists of a fixation cross (jittered at 4, 6, and 8s), a prompt screen, a movie clip, a second prompt screen, and a response screen. The task was presented using Matlab and Psychtoolbox. Each of the three runs was comprised of six experimental trials and six control trials for a total of 18 experimental trials and 18 control trials in the task.

In the experimental condition, the child sees a picture of one of the movie characters accompanied by a 5-s prompt stating, “Pay attention to this person.” The child then sees a 10-s video clip involving the character they just viewed. After the movie clip, the child is prompted with a 2-s screen depicting that character of interest paired with a prompt stating, “What would this person do next?” (*theory of mind question*). The child then sees a response screen for 6s with the options “1 – Cry,” “2 – Say Yuck,’” “3 – Smile,” “4 – Yell,” and “5 – Cover Eyes” (*theory of mind response*). Each response option corresponds to one of the five buttons on the five-button box in the child’s right hand. In this task, the ToM response condition does not have an “incorrect” behavioral response. For each scenario, the character could reasonably take several emotional responses to the situation from which the child can choose. This design better mirrors real-life experiences where several possible outcomes could occur during a social interaction.

In the social information condition, the child sees the 5-s prompt, “Count how many people you see” (*social information prompt*). The child then sees a 10-s video clip during which they count how many people they observe in the clip. Next, the child is prompted with the question, “How many people did you see?” (*social information question*), for 2s, which is followed by a 6-s response screen with the options “1 1-person,” “2 2-people,” “3 3-people,” “4 4-people,” and “5 5-or more people.”

Important to the design of this study, each movie clip is viewed twice – once as an experimental condition, *theory of mind*, and once as a control condition, *social information*; the clips are randomized. This design allows us to tightly control the stimuli such that the main difference between the conditions is what the child is being asked to do while watching the clips and responding to the prompts. In each condition, the child attends to the people in the movie clip and is then asked to think about the people viewed by either predicting behavior or counting people. Only in the experimental condition is the child asked to infer subsequent behavioral or emotional responses. Thus, our response screens – *theory of mind response* and *social information response* – comprise our conditions of interest by providing the clearest contrast of mentalizing relative to general social information.

### fMRI Acquisition Parameters

Participants were scanned on a 3-Tesla Siemens scanner at the Center for Brain Science, Harvard University. Whole-brain coverage was achieved by using 40 slices with a 3×3×3mm voxel size and a 0.5mm gap. Changes in blood oxygenation level-dependent (BOLD) MR signal were measured using a gradient echoplanar imaging (EPI) sequence (TR=2.56s, TE=30ms, flip angle=85°). Three time series with 236 volumes were obtained for each subject. We used prospective acquisition correction (PACE) on all functional runs. PACE applies an adjustment to slice acquisitions (up to 8° and 2mm) during the functional scan in order to correct for head movement throughout the scan. FMRI data were processed using SPM 8 (Statistical Parametric Mapping software; Wellcome Department of Cognitive Neurology, London, United Kingdom) in Matlab 7.4. R2007A. *Preprocessing included slice timing, realignment to the mean across all runs, co-registration of functional scans to the structural image, normalization to MNI space, and smoothing with an 8mm Gaussian kernel*. Data were high-pass filtered at 128s. The artifact detection toolbox (ART), http://www.nitrc.org/projects/artifact_detect, was used to identify outliers in movement (>2mm from the previous image) and global signal (+/− 2.5 SD from the global mean intensity signal) for each participant. Participants with more than 20% of volumes removed were excluded from analyses.

### Data Analysis

Statistical analysis was performed using the general linear model approach in order to estimate the response parameters for both ToM response and social information response. Importantly, the question and the response screens for both the theory of mind and social information conditions were modeled together (labeled as *response* in Figure 1) to reflect all neural activity involved in responding to the prompt in both the theory of mind and social information conditions. For each condition of interest (ToM and social information), hemodynamic responses were modeled to the onset of the prompt plus the response to the prompt for a total of 8s (Figure 1). Subjects’ estimates of movement outliers from ART were entered as covariates of no interest. We created the contrast *ToM response>social information response* at the individual level. A one-sample t test was conducted to examine differences between *ToM response* vs. the *social information response* at the group level. In addition to whole-brain comparisons, we also conducted region of interest (ROI) analyses. Specifically, to test *a priori* hypotheses that neural activity in ToM regions would be associated with social cognition and social behavior, theory of mind ROIs were defined from a 2016 neuroimaging meta-analysis (Molenberghs et al., 2016) of theory of mind regions. From a meta-analysis of 144 studies, Molenberghs et al. (2016) identified seven key regions related to theory of mind processing; we created regions of interest based on the seven weighted centers of each cluster which included the following: left superior temporal gyrus, right medial frontal gyrus, right superior temporal gyrus, left precuneus, right IFG, left superior frontal gyrus, and the left precentral gyrus. This method, a group rather than individually defined method, allowed us to choose functionally defined regions from the literature as our regions of interest. Previous studies focused on social-cognitive development have also used a group analysis approach to identify theory of mind regions, which supports this particular method (Richardson et al., 2018; Mukerji et al., 2019; Xiao et al., 2019). Using the MarsBar tool with SPM8, an ROI was defined as an 10mm sphere around the peak coordinates for each region. Using each ROI, a beta value was extracted for each participant’s level of neural activity in each region for the contrast ToM response>social information response. We were interested in the relationships between neural activity and the social variables (i.e., Hinting Task, RMET, and SSIS). Specifically, we ran correlations between neural activity extracted from each ROI and each of the social cognition and social functioning tasks.

## Results

The final sample included 25 children (14 females, mean age=11.24years, SD=1.39years, range 8–13years). Though prior work suggests that theory of mind changes throughout development (Gweon et al., 2012), our age range and sample size are too small to investigate developmental changes in this study. All participants completed the WISC (Wechsler, 1991) or WASI (Wechsler, 1999; *M*=115.82, SD=10.48, Range=92–135).

### Behavioral Measures

On the Hinting Task, the children performed with an average correct score of *M*=18.9 (SD=1.73, Range 13–20) out of 20. On the Mind in the Eyes Task, children performed with an average correct score of *M*=21 (SD=2.76, Range 14–28) out of 28. Parent report of social skills fell within the average range, *M*=105.71 (SD=15.53, Range 64–130). There were no significant correlations among the behavioral assessments (*p*s>0.215).

### Whole-Brain Analysis Theory of Mind>Social Information

**Figure 2:**
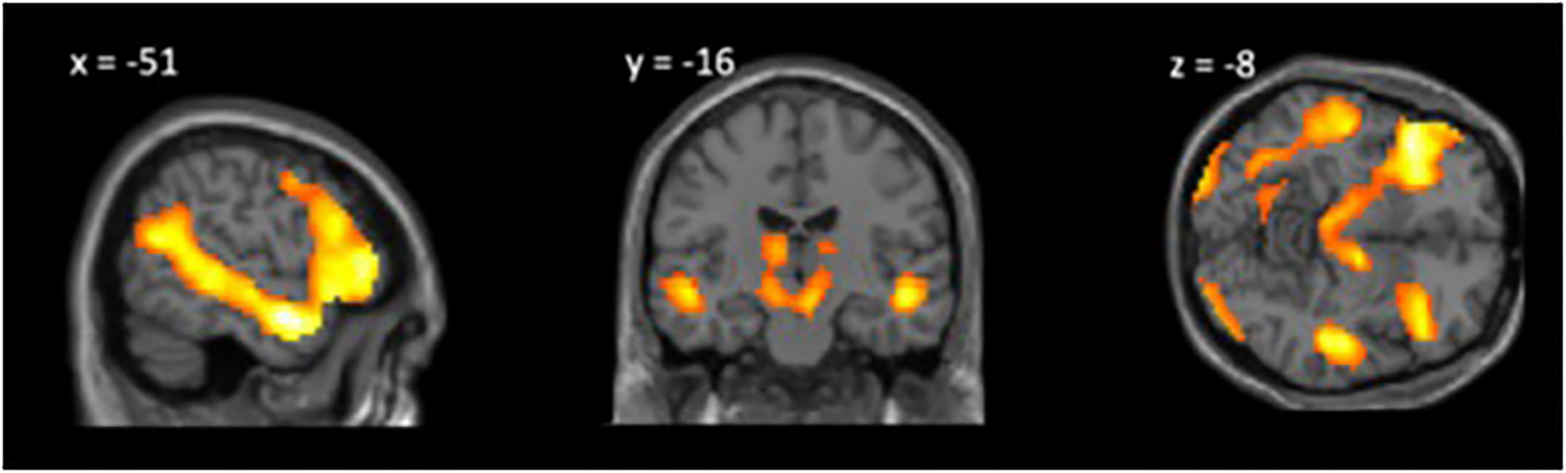
Whole-brain analysis theory of mind (ToM) Response>Social Information Response, *p*<0.05 FWE, *k*=20.

We conducted a one-sample *t* test of the *ToM response*>*social information response* contrast. The whole-brain regression was thresholded at *p*<0.05 corrected for multiple comparisons at voxel level with family-wise error with a minimum cluster size of 20 voxels (*k*=20; Table 1; Figure 2). We find significantly greater neural activity in theory of mind-related regions for the ToM relative to control condition including the left TPJ, left and right superior temporal sulcus, and left IFG.

**Table 1:** Whole-brain analysis for ToM Response>Control Response *p*<0.05 FWE, *k*=20.

Brain region	L/R	*k*	*x*	*y*	*z*	*T*	*p* at FWE (voxel level)
Superior temporal sulcus	L	1,081	−51	2	−20	9.89	<0.001
Insula	L		−27	29	−2	9.12	<0.001
Inferior frontal gyrus	L		−42	17	−8	9.03	<0.001
Superior temporal sulcus	R	115	48	−31	−5	9.28	<0.001
Superior temporal sulcus	R		51	−19	−8	7.83	0.001
Superior parietal lobule	L	69	−27	−61	46	7.93	0.001
Precentral gyrus	L	55	−45	2	46	7.93	0.001
Inferior frontal gyrus	R	141	57	29	16	7.64	0.001
Insula	R		33	17	−17	7.30	0.003
Insula	R		33	29	−5	7.09	0.004
Temporoparietal junction	R	41	66	−46	16	7.30	0.003
Fusiform gyrus	L	56	−39	−58	−17	7.03	0.005
Supplemental motor area	L	20	−6	20	46	6.32	0.019

#### Neural Activity and Social Cognition and Functioning Correlations

**Figure 3:**
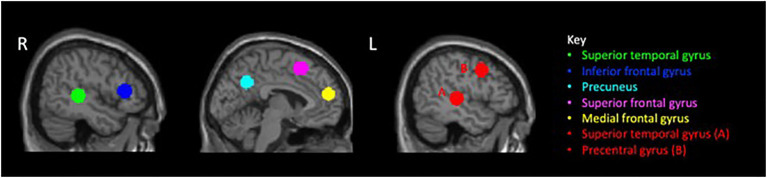
Region of interest from meta-analysis of Molenbergh (2016).

The ROIs identified in the Molenberghs et al. (2016) meta-analysis (Figure 3) were used to extract beta values for the ToM>Social information *response* contrast for each individual subject.

Correlations between neural activity in the seven ROIs and three behavioral measures of social cognition and social functioning were performed. Both the Hinting Task and the RMET task failed to meet assumptions of normality, so Spearman correlations were used. A Pearson correlation was used to test the association between neural activity in the ROIs and the SSIS (Table 2). At the statistical threshold of *p*<0.05 (uncorrected), we observe a relationship between neural activity in the right superior temporal gyrus and the Hinting Task (*r*=0.437, *p*=0.037). Additionally, However, when applying a Bonferroni correction for the number of tests (*α*=0.002), neither correlation remains significant after correction.we observe a significant correlation between the neural activity in the right IFG and the SSIS (*r*=0.435, *p*=0.049). We do not observe any significant relationships between neural activity and the RMET task (*p*s>0.409).

**Table 2:** Correlations of regions of interest and social variables.

	Hinting task	Mind in the eyes	SSIS – parent report
Left superior temporal gyrus	0.28 (−0.122, 651)	−0.16 (−0.561, 0.227)	0.32 (−0.136, 657)
Right medial frontal gyrus	−0.22 (−0.660, 0.241)	0.01 (−0.429, 0.522)	0.08 (−0.360, 0.498)
Right superior temporal gyrus/temporoparietal junction	0.44^*^ (0.035, 0.781)	0.12 (−0.378, 0.541)	0.26 (−0.189, 0.625)
Left precuneus	0.34 (−0.054, 0.648)	0.11 (−0.314, 0.546)	0.05 (−0.392, 0.470)
Right inferior frontal gyrus	0.05 (−0.435, 0.466)	−0.05 (−0.430, 0.367)	0.44^*^ (0.002, 0.729)
Left superior frontal gyrus	0.15 (−0.278, 0.521)	−0.12 (−0.487, 0.275)	0.37 (−0.072, 0.692)
Left precentral gyrus	0.17 (−0.289, 0.569)	0.05 (−0.339, 0.430)	0.40 (−0.037, 0.710)

* *p<0.05, Table contains r values and 95% confidence intervals*.

## Discussion

In this study, we aimed to (1) identify the neural correlates of theory of mind activity in children using a novel, more ecological valid fMRI task and (2) test the relationship between neural activity during a theory of mind task and the behavioral correlates of social cognition and social functioning.

First, we assessed the Movie Mentalizing Task’s ability to engage the ToM network in children. As predicted, we found that children ages 8–13 show increased activation in the ToM network in response to thinking about mental states vs. general social information. In a whole-brain analysis of the ToM condition relative to the social information condition, we find significantly greater neural activity in classic ToM regions, including the right TPJ, superior temporal sulcus, and the IFG. These findings are consistent with prior research (Gweon et al., 2012; Richardson et al., 2018; Mukerji et al., 2019) and support the use of the Movie Mentalizing Task as an experiment that taps into the ToM network. Further, this task addresses some of the concerns regarding the use of false belief paradigms with children by providing both verbal and nonverbal information for children to rely upon to understand mental states. Additionally, this task asks children to engage in mentalizing about a specific social interaction they watched unfold, applying theory of mind knowledge to understand a social situation; this aspect of our task differs from other tasks that may lack dynamic social interaction (e.g., Borbas et al., 2021). Moreover, as an additional benefit, a movie task may be easier and more engaging for children in the scanner (Jacoby et al., 2016; Richardson et al., 2018).

Of note, we do not find neural activity in the MPFC in our theory of mind relative to social information condition. The MPFC is a region that has been consistently identified as important in adult studies of theory of mind (Shallice, 2001; Molenberghs et al., 2016), but there are mixed findings in children with the MPFC sometimes recruited for ToM (O’Nions et al., 2014) and in other times not (Mukerji et al., 2019). Some research has found age-related changes in activation in the MPFC from childhood to adolescence, whereas these changes were not noted in other ToM regions like the superior temporal sulcus (Moriguchi et al., 2007). It is possible that given the young age of our participants the MPFC did not differentiate the social information in the control condition relative to the specific ToM information in the experimental condition. Perhaps greater specificity would come with continued maturation. Alternatively, the MPFC may have responded more broadly to *people*, rather than to specific theory of mind information, thus having a similar level of activity in both our theory of mind and social information conditions. Previous research suggests that the MPFC is associated with making judgments about similar others (Mitchell et al., 2006) as well as attending to the processing of socially relevant information that is important to the individual (Krienen et al., 2010); these findings support the idea that the MPFC may respond similarly to our two conditions.

A second goal of this study was to test the relationship between functional activity in ToM regions and behavioral correlates of social cognition and social functioning. We hypothesized that greater neural activity in ToM regions would be related to better performance on social-cognitive and social functioning tasks. Investigating the association between neural activity in our ROIs and social-cognitive performance, we found a positive relationship between neural activity in the right posterior superior temporal gyrus and the Hinting Task. Additionally, we found a positive relationship between neural activity in the right IFG and parental report of social functioning, with greater activity associated with better social functioning. The IFG is consistently activated across ToM tasks (Shamay-Tsoory et al., 2009; Dal Monte et al., 2014) and is often discussed as part of the mirror or simulation system (Molenberghs, 2012). Additionally, the IFG is commonly associated with affective, emotion understanding, relative to cognitive, processing beliefs and thoughts, in ToM (Molenberghs et al., 2016). Our finding is consistent with the idea that the IFG is implicated in experiencing and inferring the feelings of others, a skill that is critical to successful functioning. In contrast to a false belief task, a cognitive ToM task, which focuses on beliefs, the movie mentalizing task could be considered an affective ToM task with a focus on emotion inference. Previous research suggests important differentiation in neural processes and behavioral functioning in affective vs. cognitive theory of mind tasks (Sebastian et al., 2012), and our study supports the importance of testing affective theory of mind. The possible relationship between neural activity and the parent-reported social skills demonstrates a unique brain-behavior relationship. Different than an association between neural activity and theory of mind performance, this study shows that neural activity may be linked to real-world behavior, which begins to give us insight into how social functioning may be affected by neurobiology. This research expands upon previous studies that have also used movies as theory of mind stimuli (e.g., Richardson et al., 2018; Richardson and Saxe, 2019) because we test this brain-behavior relationship. The brain-behavior relationship we see between neural activity in ToM regions and children’s real-world social functioning is consistent with findings in adults. For example, in healthy adults, greater activity in ToM regions is related to better daily social functioning (Dodell-Feder et al., 2014). Our findings, though nonsignificant after correcting for multiple comparisons, suggest that this brain-behavior relationship is similar in children and adults. This finding should be replicated with larger samples. Our results help establish the link among the social brain, social cognition, and social functioning, which is a critical relationship to understand its development in typically developing children and its potential dysfunction in individuals with neurological and psychiatric disorders (Kennedy and Adolphs, 2012).

Though we see a possible brain-behavior relationship with specific ToM regions, we do not see it across all ToM regions (e.g., left superior temporal gyrus) or across all tasks (e.g., RMET). There are several possibilities to explain these findings. The Hinting Task is a relatively easy ToM task for typically developing children, and most of our participants performed extremely well, potentially a result of having children with above-average IQs as the sample, with little variation, suggesting a possible ceiling effect. Of note, we found no relationship between neural activity in ToM-related regions and the RMET. One potential reason for this lack of relationship may be that the child version, like the adult version of the RMET, might be more sensitive to specific sociocultural factors like education, ethnicity, and race, as much as social-cognitive ability (Dodell-Feder et al., 2020). While these associations have not been tested in the child version of the RMET, some of the same problems of the adult version exist in the child version (e.g., difficult vocabulary, ethnically homogenous stimuli). These potential problems could explain why we find no relationship between neural activity in ToM regions and performance on the RMET. Moreover, these concerns regarding the RMET further support the need for more valid tasks like the Movie Mentalizing Task. Additionally, we do not see a relationship across all ToM regions with social behavior; it is possible that our measure of social behavior is too broad and focuses more on global aspects of social functioning rather than specific social behaviors.

This study is not without limitations. First, our brain-behavior correlations were not significant after controlling for multiple comparisons; thus, the brain-behavior relationships should be interpreted cautiously. Additionally, while our task is not heavily reliant on language and inhibitory control, we note that our theory of mind response condition screen provides the participant with both a face picture and emotion-related words, which by themselves might tap in the theory of mind network, whereas the social information response condition screen has no face or emotion-related words. Thus, our task does not completely control for all differences in the conditions. These limitations may affect the generalizability of our study. In addition, we do not see the expected relationship between the behavioral measures of social cognition (e.g., Hinting Task and RMET). It is possible that the ceiling effect on the Hinting Task affected this relationship. Future studies should include more complex theory of mind tasks (e.g., a faux pas task) created for typically developing children. While we attempted to pick a movie stimulus that many children would not have seen given its original date (1994), it is possible that some children may have previously viewed this movie and that might have affected the results. Finally, we did not take a post-test of children’s knowledge of the vocabulary in the RMET, and thus, we do not know whether variation in language ability might have affected the outcome.

Further research is necessary to continue to elucidate the nature of this brain-behavior relationship, as well as to look at how it changes across the lifespan, as previous research suggests that there are developmental changes in these regions (Gweon et al., 2012). Future studies could have a larger sample, investigate associations with other demographic variables such as socioeconomic status, and provide social functioning reports from the children and/or their teachers. It is imperative that we identify the mechanisms that help support real-world social functioning if we want to better understand both typical and atypical social development.

Theory of mind development is pivotal to children’s social successes. Research has begun to map the neural network of ToM throughout childhood into young adulthood. This task contributes to that literature by using a novel affective theory of mind task that robustly recruits the social brain regions. This task potentially improves upon previous tasks by having dynamic social stimuli and reducing the burden of the cognitive complexities in other theory of mind tasks (e.g., faux pas task). Additionally, whether neural activity in these regions is related to social functioning in children has rarely been tested, and this study fills that gap in the literature. This work suggests a possible brain-behavior relationship, which can provide insight into the development of social-cognitive processes like theory of mind in typically and atypically developing children; this insight could aid in intervention efforts with individuals who have developmental delays or impairments in these abilities.

## Data Availability Statement

The raw data supporting the conclusions of this article will be made available by the authors, without undue reservation.

## Ethics Statement

The studies involving human participants were reviewed and approved by Harvard University Institutional Review Board. Written informed consent to participate in this study was provided by the participants’ legal guardian/next of kin.

## Author Contributions

SHL: study conceptualization, task design, data collection, data analysis, manuscript preparation, and revisions. CM: task design, data collection, data analysis, manuscript preparation, and revisions. DD-F: task design, manuscript preparation, and revisions. AR: data analysis, manuscript preparation, and revisions. CH: study conceptualization, task design, data collection, data analysis, manuscript preparation, revisions, and supervision. All authors contributed to the article and approved the submitted version.

## Funding

The project was supported through funding from the Sackler Scholar Programme in Psychobiology (SHL) and the Richmond Fellowship from the Center on the Developing Child at Harvard University (SHL).

## Conflict of Interest

The authors declare that the research was conducted in the absence of any commercial or financial relationships that could be construed as a potential conflict of interest.

## Publisher’s Note

All claims expressed in this article are solely those of the authors and do not necessarily represent those of their affiliated organizations, or those of the publisher, the editors and the reviewers. Any product that may be evaluated in this article, or claim that may be made by its manufacturer, is not guaranteed or endorsed by the publisher.
